# Phase transition and thermal stability of epitaxial PtSe_2_ nanolayer on Pt(111)

**DOI:** 10.1039/d0ra04346j

**Published:** 2020-08-20

**Authors:** Yongfeng Tong, Meryem Bouaziz, Hamid Oughaddou, Hanna Enriquez, Karine Chaouchi, François Nicolas, Stefan Kubsky, Vladimir Esaulov, Azzedine Bendounan

**Affiliations:** Synchrotron SOLEIL - L'Orme des Merisiers Saint-Aubin - BP 48, 91192 Gif-sur-Yvette Cedex France azzedine.bendounan@synchrotron-soleil.fr; Institut des Sciences Moléculaires d’Orsay, UMR 8214, Université Paris-Sud, Université Paris-Saclay 91405 Orsay Cedex France; Département de Physique, Université de Cergy-Pontoise 95031 Cergy-Pontoise Cedex France

## Abstract

This work relates to direct synthesis of the two-dimensional (2D) transition metal dichalchogenide (TMD) PtSe_2_ using an original method based on chemical deposition during immersion of a Pt(111) surface into aqueous Na_2_Se solution. Annealing of the sample induces significant modifications in the structural and electronic properties of the resulting PtSe_2_ film. We report systematic investigations of temperature dependent phase transitions by combining synchrotron based high-resolution X-ray photoemission (XPS), low temperature scanning tunnelling microscopy (LT-STM) and low energy electron diffraction (LEED). From the STM images, a phase transition from TMD 2H-PtSe_2_ to Pt_2_Se alloy monolayer structure is observed, in agreement with the LEED patterns showing a transition from (4 × 4) to (√3 × √3)R30° and then to a (2 × 2) superstructure. This progressive evolution of the surface reconstruction has been monitored by XPS through systematic de-convolution of the Pt4f and Se3d core level peaks at different temperatures. The present work provides an alternative method for the large scale fabrication of 2D transition metal dichalchogenide films.

## Abstract 2

LEED, STM and XPS techniques were used to systematically study a temperature-dependent phase transition on a PtSe_2_ film grown on the surface of Pt(111) by a chemical deposition method.

## Graphical abstract

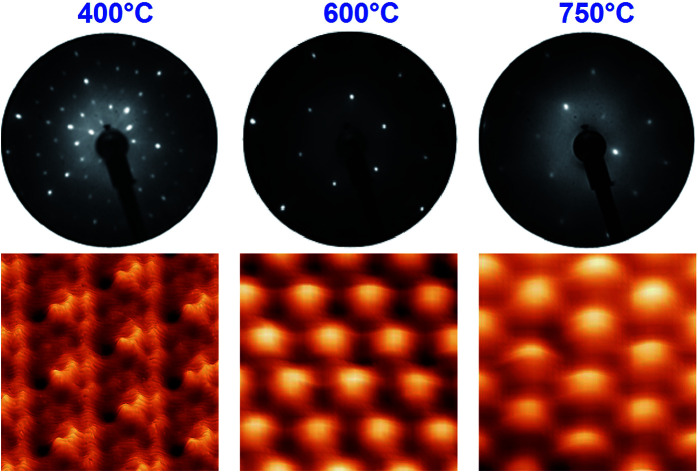

## I. Introduction

Two-dimensional (2D) nano-materials have been extensively studied over the past decade and are now being considered as the next generation of materials for future high-technology applications. In particular, the rapid advances in graphene and silicene research^1–5^ have led to considerable interest in other types of 2D systems such as germanene, phosphorene, boron nitride and transition metal dichalchogenide (TMD) layers.^6–10^ In addition to chemical composition, atomic arrangement and dimension proved to be one of the most important parameters in determining film properties and device performance. 2D layer materials, especially in the monolayer limit, have aroused enormous attention because they exhibit interesting novel properties that are rather different from those of the bulk phase. Among the ideal candidates, TMDs have versatile physical and chemical properties that offer great potential for exploration in areas such as energy storage, nano-electronics and sensor technologies. The TMDs' family is described with the common formula MX_2_, where M stands for a transition metal (M = Mo, W, Nb, Ta, Ti) and X for the chalchogenide element, *i.e.* S, Se or Te. MX_2_ forms either the 1T- or 2H-structure (with the sub-X layers 180°- or 0°-rotated with respect to each other) without surface dangling bonds. The former is of a metallic character while the latter has a semiconductor nature. Depending on the metal and the chalcogen involved in the growth process; their electrical properties span the range from semiconducting to superconducting. For instance, phase transitions of 1T to 2H in 2D TMDs tend to occur at elevated temperatures or by simple lithiation.^11^ Many of the TMDs require chemical modifications to make them suitable for a wide variety of applications.

In addition, MX_2_ can exhibit peculiar transitions like metal–insulator transition, or superconductivity at low temperature. The most studied TMDs are those based on S and Se atoms. These two chemical elements are also involved in functionalization activity of the so-called organic self-assembled monolayers (SAMs) where the chalcogen atom of the head group provides the bond with the metal substrate.^12,13^ Intensive effort has been dedicated to the study of sulfur interaction with Au, Ag and Cu surfaces, focusing on the formation of self-assembled monolayers and its behavior under various environments. These studies range from analyzing atomic S to molecules with chain structure (alkane) or aromatic molecule (thiols, dithiols, thiophene). The interaction between the S headgroup and metal surfaces plays an important role in determining the characteristics of the organic–metal hybrid system, including film structure, orientation, bond state. The layered MoS_2_, PtS_2_ are also the first 2D TMD materials that have been studied for their physical and chemical properties.^14,15^

Perhaps selenium (Se) has attracted less interest; it nevertheless has good photovoltaic and photoelectric properties, which explains why Se-based compounds are being successfully introduced in the fields of Lithium Ion Battery (LIB), solar cells, detectors and lasers.^16–18^ Attention has been paid to the Se-based TMD materials like MoSe_2_, WSe_2_ and recently PtSe_2_. In particular, a PtSe_2_ monolayer was epitaxially grown on a clean Pt(111) surface.^19^ Upon annealing at 250 °C, a (3 × 3) superstructure was observed by low energy electron diffraction (LEED). An octahedral structure was proposed according to the observation from the scanning transmission electron microscopy (STEM) image of the film cross section.^19^ The bulk PtSe_2_ is a semimetal with zero band gap,^20,21^ however the PtSe_2_ monolayer has a band gap of 1.2 eV confirming theoretical predictions to this effect.^22–24^ Previously, PtSe_2_ has been used as a photocatalytic material in graphene nano-composites.^25^ In addition, its potential for hybrid electronic devices has also been demonstrated through the example of a high-performance gas sensor and photo-detector, as well as a photovoltaic cell.^26^

The PtSe_2_ monolayers have been produced by vacuum evaporation of Se on a Pt (111) substrate or by what the authors of ref. 26 called thermally assisted conversion (TAC) in which a Pt thin film grown on a silicon substrate (Si/SiO_2_) was exposed to Se vapor in an Ar–H_2_ flow. In the first case the selenized Pt(111) substrate was heated to 200 °C and then 270 °C leading to PtSe_2_ formation as characterized by LEED, X-ray photoemission spectroscopy (XPS), scanning tunneling microscopy (STM) and angle resolved photoemission spectroscopy (ARPES) and in the TAC method best results for PtSe_2_ several layer formation from a Pt film was found to be at 400 °C. At low temperature full selenization was not obtained as monitored by XPS. This direct selenization method is akin to formation of PtO_2_ single layers by direct oxidation of Pt reported some years ago.^27–29^ In another study the PtSe_2_ single crystal nanosheet was fabricated with H_2_PtCl_6_ and Se as a precursor by Wang *et al.*^30^ The p-type transport property with mobility larger than 7 cm^2^ V^−1^ S^−1^ for the PtSe_2_ nanosheet was obtained and the electronic property of the MoS_2_/PtSe_2_ p–n junction was also investigated.^30^

In this paper we report another way for the elaboration of the PtSe_2_ layer consisting of a direct selenization of Pt(111) by liquid phase immersion in a Na_2_Se solution, followed by annealing procedure under ultrahigh vacuum (UHV), as was done in our earlier works on selenization of Ag, Au and Pd.^31,32^ From a device manufacturing perspective, the thermal stability of PtSe_2_ is highly important. We have therefore studied this effect and present here the role of the temperature in the formation PtSe_2_ monolayer and the resulting modifications in the electronic structure. Results obtained by high-resolution XPS, low-temperature STM (LT-STM) and LEED will be presented and discussed in the remainder of the paper.

## II. Experiment

### II.1. Sample preparation

The Pt(111) single crystal was acquired commercially from the Surface Preparation Laboratory in the Netherlands. *In situ* preparation of this crystal was made by multiple cycles consisting of Ar^+^ sputtering followed by annealing at 600 °C in O_2_ pressure of about 5 × 10^−8^ mbar, before flashing at high temperature *T* ∼ 900 °C in ultra-high vacuum (10^−10^ mbar). The surface cleanliness was verified by high-resolution XPS showing the absence of carbon and oxygen core level peaks. The quality of the surface crystallinity was characterized by LEED.

For the selenization of the Pt(111) surface, commercial Na_2_Se powder was purchased from Sigma Aldrich and was used as received without further purification. An aqueous NaSe_2_ solution was prepared in a glove box under nitrogen (N_2_) atmosphere, by dissolving 1.25 mg of the selenide in 100 ml of Milli-Q water. Under the same conditions, the selenization operation was performed by immersing the clean Pt(111) surface into the 100 ml of the prepared NaSe_2_ solution for a duration of about 2 min. The resulting surface was then thoroughly rinsed with Milli-Q water and dried under N_2_ flow before reintroducing into the UHV setup. This allows a spontaneous formation of a selenized layer on the surface.^33^ Therein, the Se atoms react strongly with the Pt surface, while the Na atoms are removed after the rinsing with water. Afterwards, the measurements were performed on the sample as-prepared and after being annealed in UHV at different temperatures. The latter were controlled by a thermocouple located close to the sample. Bearing in mind that the handling of Se is extremely dangerous, this method would be the ideal way to develop Se-based systems at large scale with great safety. It can be compared to other chemical syntheses such as solvothermal and sol–gel, which are mostly employed for the elaboration of metal oxide and metal chalcogenide nanoparticles or nanostructure products.^34–38^ However, the key step of the method discussed here is the *in situ* annealing of the sample at appropriate temperature under UHV conditions that allows desorption of the contamination species and crystallization of the film structure. Another well-known deposition technique is Molecular Beam Epitaxy (MBE)^39,40^ but for Se, one needs a dedicated evaporator and a heavy pumping system.

### II.2. Characterization

The high-resolution photoemission experiment was performed using synchrotron radiation at TEMPO beamline of Synchrotron SOLEIL, France. The measurements were made with a pass energy of 50 eV giving an energy resolution better than 50 meV (spectrometer and beamline). Different photon energies were used and we have selected an excitation energy 100 eV higher than the binding energy of the core level of interest. This was done to ensure a good surface sensitivity by obtaining a reasonable photoionization cross section and to avoid high secondary electron background. The energy scale was also calibrated with the Au4f_7/2_ on a clean Au(111) surface at the corresponding photon energies. The experimental binding energy calibration error for the reported core level binding energies (CLBE) is estimated to be of ±50 meV. LT-STM measurements were conducted on an Omicron STM station at ISMO-CNRS laboratory, Paris-Saclay University. While the LEED and XPS measurements were made at room temperature, the STM images were recorded at low temperature (∼77 K) with liquid N_2_ cooling in order to reach the atomic resolution, which was mandatory for the present study. No further treatment was applied to the STM images.

The fitting of the photoemission spectra was performed using the CasaXPS software.^41^ After a proper Shirley background subtraction, the Pt4f features were fitted with the so-called Doniach–Sunjic line-shape, while for the Se3d levels, we have used a Voigt type line-shape (convolution of a Gaussian and Lorentzian distributions). Spin–orbit splitting of 3.4 eV between Pt4f_7/2_ and Pt4f_5/2_ levels and of 0.86 eV between Se3d_5/2_ and Se3d_3/2_ were considered.

## III. Results description

### III.1. LEED and STM measurements

**Fig. 1:**
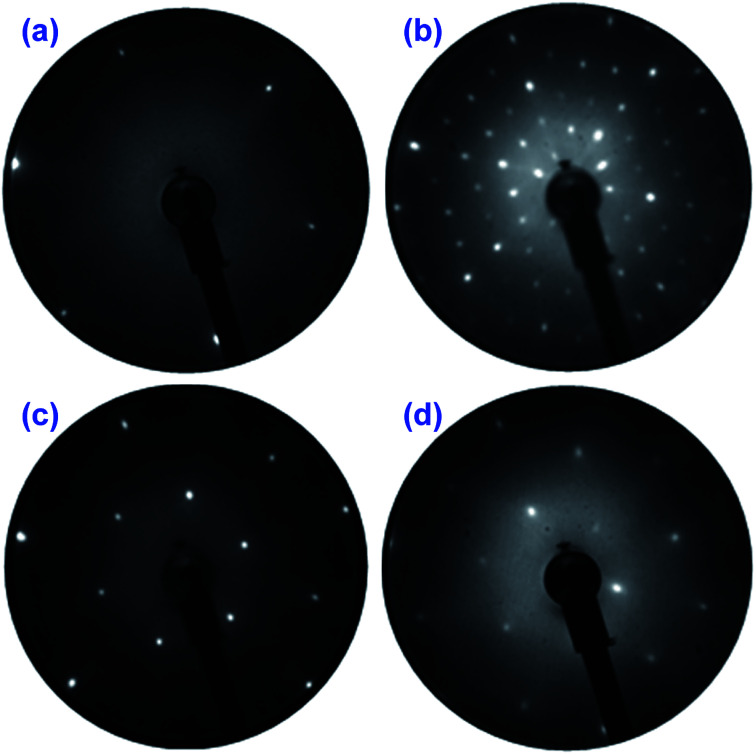
Low energy electron diffraction (LEED) patterns of (a) clean Pt(111) and (b–d) of Se film on Pt(111) annealed respectively at 400 °C, 600 °C and 750 °C. The LEED patterns were recorded at an energy of 80 eV.

The LEED results obtained on the PtSe_2_ surface being annealed at different temperatures are illustrated in Fig. 1, and for comparison a LEED pattern of clean Pt(111) is also shown. The measurement was performed at different positions and usually an identical LEED pattern was obtained, which indicates an ordered and uniform PtSe_2_ film over the entire sample surface. Upon annealing at 400 °C for 10 minutes, a (4 × 4) pattern with respect to the Pt lattice was clearly observed, as seen in Fig. 1b in comparison to the hexagonal lattice in reciprocal space measured on clean Pt(111) surface at the same energy, shown in Fig. 1a. The same result was obtained by Wang *et al.* using a different synthesis method.^30^ No change in the LEED pattern was observed until 600 °C, where a (√3 × √3)R30° pattern appeared, given in Fig. 1c. At higher annealing temperature 750 °C, another superstructure develops and the LEED pattern in Fig. 1d indicates (2 × 2) reconstruction with respect to the Pt(111) lattice.

**Fig. 2:**
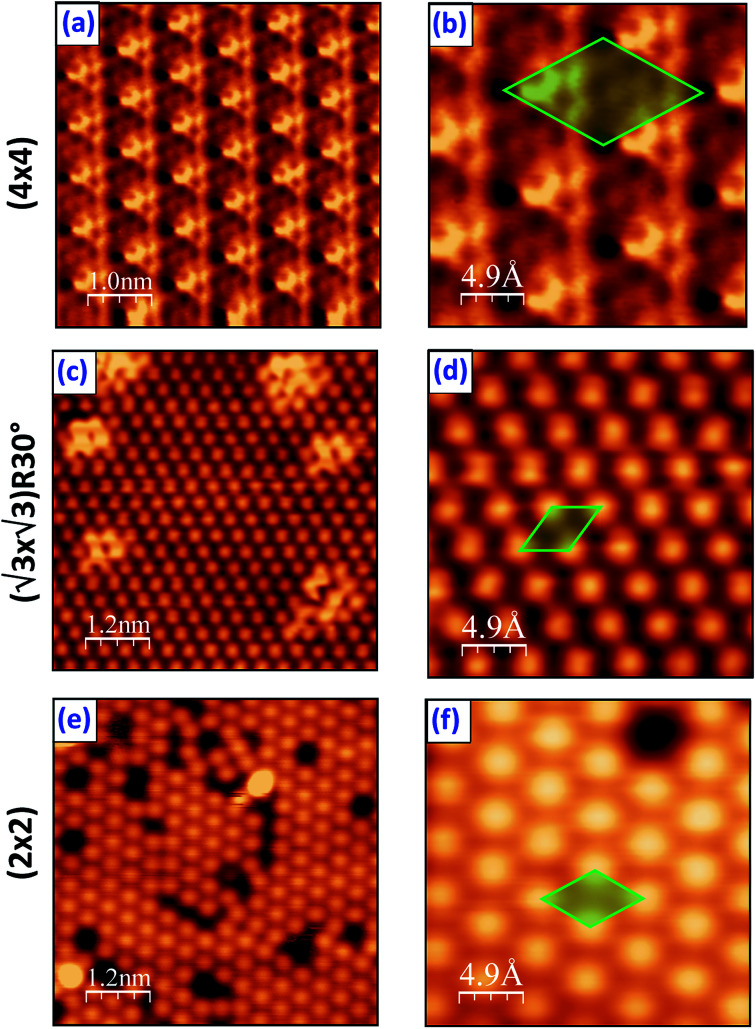
Atomic resolution STM images obtained on Pt(111) covered by Se film followed by annealing at: (a, b) 400 °C, (c, d) 600 °C and (e, f) 750 °C, respectively.

LT-STM was employed to examine the surface structure of the PtSe_2_ films at the atomic scale. After annealing at 400 °C, a well-defined moiré pattern of the PtSe_2_ was observed due to its mismatch with the Pt(111) surface (Fig. 2). The periodicity of the moiré pattern is approximately 11 Å, which is nearly 4 times the lattice constant of Pt(111). A hexagonal honeycomb arrangement is clearly visible in a zoomed area from the moiré pattern (Fig. 2b), for which a 2H trigonal prismatic coordination can be proposed. The atomic arrangement is also consistent with the TEM results on PtSe_2_ or other TMD systems in literature.^42–46^ The structure changed dramatically after annealing the sample at 600 °C as indicated in Fig. 2c and d, the previous 2H phase is replaced by a new (√3 × √3)R30° superstructure. This latter is a strong indication of formation of a surface alloy, type Pt_2_Se. One notices the presence of several bright domains on the surface that may originate from insufficient heating. Similar domains were observed by Zheng *et al.*, and were interpreted as Se-rich antisite defects.^47,48^ After a further annealing at 750 °C, the unit cell of the Pt_2_Se undergoes a rotation of 30° compared with the (√3 × √3)R30° structure at 600 °C, resulting in a (2 × 2) pattern with respect to the Pt lattice, as seen in Fig. 2e and f. For the (2 × 2) structure, one observes a large density of defects on the surface which is consistent with the LEED pattern showing high-background and diffusing diffraction spots.

### III.2. Photoemission measurements

**Fig. 3:**
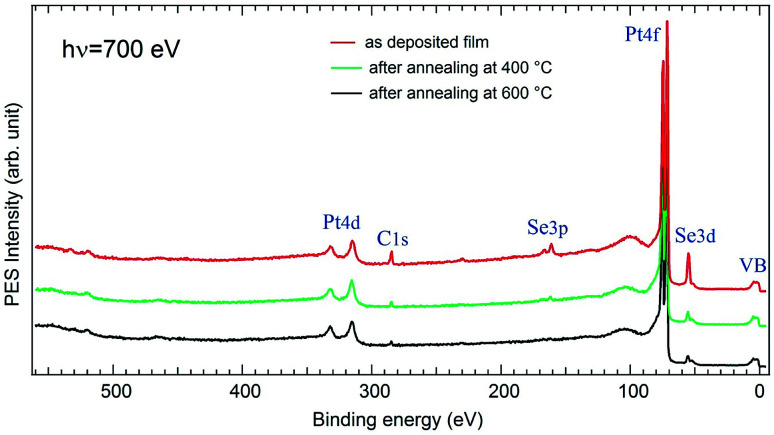
Overview spectra measured with *hν* = 700 on Pt(111) covered by as-grown Se film, after annealing at 400 °C, and after further annealing at 600 °C, respectively. Several features are observed like the Pt4f centred at 72 eV, Se3d at 54 eV, Se3p at 162 eV. Only a tiny C1s peak at 285 eV and almost no O1s structure at 531 eV are seen. These contaminations come from the air atmosphere during the film deposition where the substrate was incubated into the solution. Upon annealing the O and C are significantly attenuated. They shall not affect the PtSe_2_ formation so we don't talk about it in the following.

Fig. 3 shows an XPS overview spectrum measured on a freshly deposited Se film compared to those after annealing at 400 °C and at 600 °C, respectively. Several core level features are present like the Pt4f at 71 eV, the Se3d at 54 eV, and Se3p at 162 eV. One observes also a tiny C1s peak at 285 eV and almost no structure of the O1s at 531 eV. Such low contamination may come from uncontrolled exposure to air during the film preparation when the substrate was incubated in the Na_2_Se solution, although this operation was made under N_2_ flow condition. One notices also the absence of Na core level peaks. After annealing, the C intensity is considerably attenuated and that of O disappears almost completely. This proves a relative cleanliness of the surface and demonstrates that our method for the preparation of PtSe_2_ films can be generalized and widely used in different research and technological fields.

**Fig. 4:**
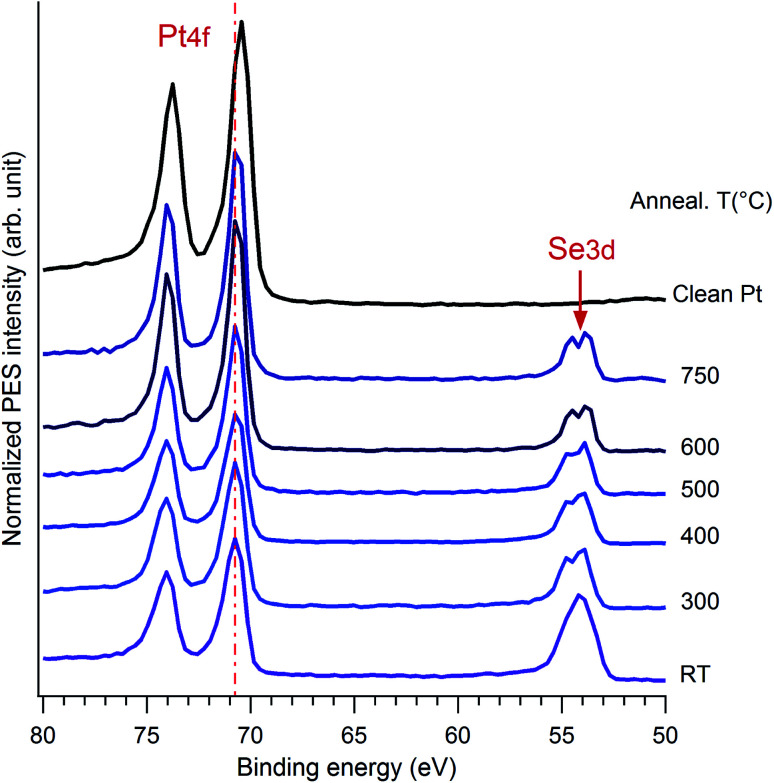
Set of XPS spectra measured on Pt(111) covered by Se layer and showing the evolution of the Pt4f and Se3d core level peaks as function of the annealing temperature, in comparison with spectrum obtained on pristine Pt(111). The data were recorded at 260 eV photon energy at normal emission.

Fig. 4 illustrates the evolution of photoemission spectra as a function of annealing temperature, together with the one measured on a clean Pt surface, at *hν* = 260 eV. At this energy, we mainly probe the Pt4f and Se3d core levels located around 71 eV and 54 eV, respectively. A significant difference in the peak intensity between these two levels is due to many parameters. In particular, one should take into consideration the respective cross section of excitation of the Se and Pt levels, as well as the electron mean free path. The measurement parameters like the transmission curve of the electron spectrometer also have an influence. Note that at 260 eV photon energy, the Se3p cross section is an order of magnitude smaller than that of Se3d and hence peaks due to the latter state are much more prominent.^49^ Also, the cross section of the Pt 5p (lying at about 51.5 eV) is about 30 times lower than of Pt4f and about 50 times lower than that of Se3d, thus it is not visible in the spectra presented here.^49^ This is why hereafter we refer solely to the CLBEs of the lower lying spin orbit components: Pt4f_7/2_ and Se3d_5/2_ and to the ratio between their peak intensities.

As a function of temperature, the first significant change, especially for the Se3d level, is observed when the film is heated to 300 °C. This is largely due to desorption of oxygen and carbon atoms, as well as bulk like Se atoms from the surface and the rearrangement of the surface structure. Then, an intensity balance occurs in the range of 300 °C to 600 °C and the spectral weight of Pt4f increases while that of Se3d decreases. This is a clear indication of desorption of the Se top-layers from the surface, allowing the reduction of the film thickness. No significant change is observed after annealing at temperature above 600 °C.

**Fig. 5:**
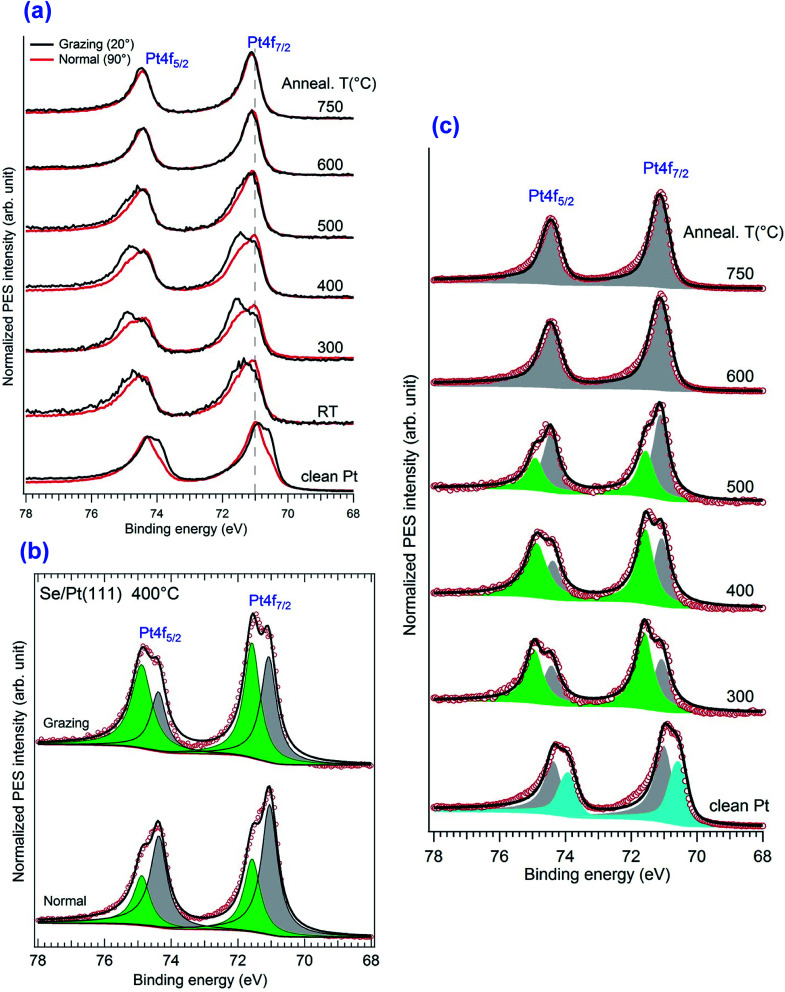
XPS spectra of the Pt4f features for normal and grazing emission on pristine and selenized Pt(111) surfaces. (a) Superposed normal and grazing emission spectra, (b) fitting of spectra obtained at 400 °C annealing temperature and (c) global view of the fitting of spectra obtained Grazing emission and at different annealing temperatures.

Now we take a closer look at the details of the XPS data. Upon annealing, we see a substantial change in the spectra of Pt4f and Se3d core levels, the details of which are given in Fig. 5. The angles indicated here correspond to the one between the emitted electron captured by the analyser and the surface plane. Thus 90° means the normal emission while the 20° is the grazing emission case. One can distinguish that the angular dependence only appears in the range of 300–400 °C of annealing, while it disappears at higher temperature, above 600 °C. A better understanding of the angular dependence behaviour required a fitting of the spectrum with two different components corresponding to the bulk contribution and the surface one, the latter of which in our case should be assigned to the Pt in the PtSe_2_ films as reported previously in literature. The surface component in the clean Pt spectrum was also proved by other authors.^27,28^

As mentioned above, Doniach–Sunjic line-shapes were used to fit the Pt4f spectra, after subtraction of a Shirley background. Starting from the clean substrate, the Pt_7/2_ surface and bulk components are located at 70.5 eV and 71.1 eV, respectively. No oxidation features located at 72 eV or a hump at 76.9 eV was observed.^50^ The surface component is shifted to 71.4 eV upon initial selenization, which was similar to the PtO_2_ case.^27,28^ The two components co-exist from 300–500 °C and the PtSe_2_ component disappeared totally at 600 °C while the Pt bulk contribution increases slightly. In accordance to the overview results, the intensity ratio between the surface component and the bulk one kept decreasing upon annealing. The most prominent angular dependence at 400 °C was extracted in Fig. 5b. Measurements show that at grazing emission the higher energy component is considerably enhanced, indicating that it corresponds to Pt atoms in the vicinity of the surface. The general features of these spectra are similar to previous reports of oxidation of Pt^27,28^ and of the recent work on PtSe_2_ nano-layer formation.^26^ The low energy Pt_7/2_ contribution lying at 71.1 eV is associated with the Pt^0^ of the bulk covered by the sandwich-like SePtSe structure. The higher energy feature could be assigned to Pt^2+^, which was also given in PtNi system.^51^ Yim *et al.*, gave the corresponding peak position at about 72.3 eV after annealing to 400 °C but for the case of a trilayer PtSe_2_ film^26^ the position of this peak evolves in case of oxidation. Thus, Miller *et al.*, reported a CLBE of the highly oxidized Pt film of about 72.1 eV and a position of about 71.6 eV for lower oxygen exposure, although they consider a OPtO structure, which evolves from wire like formation to a monolayer film.^28^ They suggested that they may have up to two PtO_2_ layers in case of high oxidation. Possibly the CLBE for a single PtSe_2_ layer on Pt(111) is lower than in the multilayer case. We shall return to this point in the following. Upon annealing of the sample at 600 °C, the spectroscopic Pt^2+^ feature at 71.4 eV vanishes completely. This result is a strong evidence of the disappearance of the 2H phase, *i.e.* of SePtSe sandwich structure and occurs to be in great agreement the LEED and STM outcome suggesting the formation of a single monolayer of Pt_2_Se surface alloy. Interestingly, when the remaining surface was heated at temperature higher than 750 °C, the surface component of the clean Pt, which previously shifts to a higher binding energy of 71.1 eV in the Se containing structure, started to appear again at binding energy 70.6 eV, which is assigned to the surface component of clean Pt(111). This means that the Pt_2_Se has vanished through desorption process of the Se atoms induced at high temperature.

**Fig. 6:**
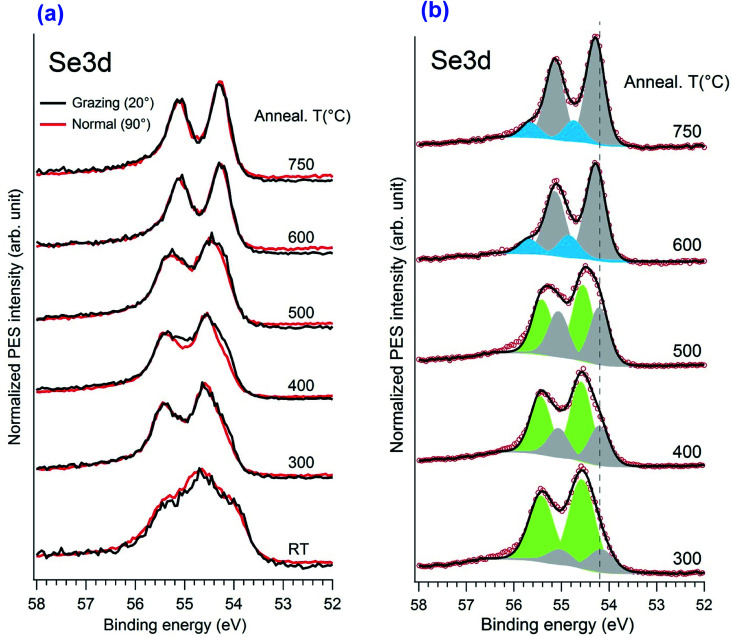
(a) XPS spectra illustrating the evolution of the Se 3d core level peaks as a function of annealing temperature for normal and grazing emission geometries. (b) Fitting of Se3d spectra at different annealing temperatures measured at normal emission.

The evolution of the Se3d core level *versus* annealing temperature is illustrated in Fig. 6. After initial selenization, the spectrum shows multi-component Se3d_5/2_ peaks with a shoulder at 54.0 eV and a main peak at 54.6 eV. In previous studies of Se adsorption from Na_2_Se on Au and Cu it was noted that initial adsorption leads to appearance of selenium corresponding to metal selenide, chemisorbed Se atoms, and also to some ring or bulk like components.^31,33^ Because of the very low melting point of bulk Se (tabulated value of 221 °C) one can suppose that any bulk like Se would be rapidly eliminated upon heating, leaving more strongly bound components. The structures are quite similar when annealing at 300 °C to 500 °C, more or less resolving two components. But an angular dependence is observed only for the 400 °C and 500 °C cases, although is less visible for 500 °C. The detailed fitting of the Se3d spectrum is given in Fig. 6 with the Voigt fitting procedure after the Shirley background subtraction. The fitting parameters were constrained to reduce the uncertainty: the Se3d spin orbital split of 0.86 eV and a ratio of 2/3 were utilized. All the Voigt width was kept at 0.55 eV. As indicated here, from 300 °C to 500 °C the spectra were de-convoluted with two main doublets with the 3d_5/2_ CLBE locating at 54.2 eV and 54.6 eV, respectively. In earlier work, Yim *et al.*, reported a somewhat broad and structure-less Se 3d_5/2_ spectrum with a CLBE given to be 54.6 eV for a trilayer film. Wang *et al.* report a structured spectrum with a maximum (Se3d_5/2_) at about 54.7 eV and a shoulder at 54.3 eV at 200 °C. At 270 °C they present a broad doublet with a 3d_5/2_ CLBE of 54.3 eV for the monolayer film. In our case it was close to the monolayer structure. The angular dependence at 400 °C, in which the 54.3 eV component became more predominant at grazing angle, also clearly indicated a SePtSe trilayer structure with the 54.2 eV peak assigned to the upper Se atoms of the trilayer. The spectroscopic feature positioned at 54.6 eV is associated with the down Se atoms located between the bulk Pt atoms and the Pt atoms of the SePtSe trilayer.

Significant change of the peak shape happened only after annealing at temperature higher than 600 °C. The upper Se component shifts only slightly to 54.3 eV while the higher energy component vanishes totally and no angular dependence is observed. Moreover, the ratio in the XPS spectral weight between the Se3d and Pt4f peaks decreases significantly, which is consistent with the fact that only one Se–Pt monolayer exists on the surface. One notices the occurrence of a new feature with low spectral weight at a binding energy of 54.9 eV (blue peak in Fig. 6), which is most likely attributed to the defect regions rich in Se atoms.

## IV. Discussion

We do not discuss the properties of the film below 300 °C because no LEED patterns or STM images could be observed, indicating either the non-homogeneous structure of the surface below this temperature or the presence of a considerable amount of Se deposit. The second reason is more likely, as it could explain the similarity of Se3d and Pt4f spectra to those obtained after annealing at 400 °C. We would mainly focus on the 400–600 °C phases, as they offer us interesting structural information. The surface displayed a good SePtSe trilayer after the reconstruction at 400 °C. The LEED showed a (4 × 4) structure of the PtSe_2_ layer with respect to the Pt(111) surface lattice. LT-STM provided a moiré pattern of the PtSe_2_, which corresponds to the model of trigonal prismatic coordination proposed elsewhere.^19^ This was commonly seen in the MoS_2_ and other metal chalchogenide structure.^42,52^

Phase transition in TMD systems represents an interesting aspect to investigate. Transition from the 2H phase to a metallic 3R phase was reported on exfoliated MoS_2_ nanosheets.^53^ Besides, a 1T to 2H phase transition was observed in MoS_2_ due to the intercalation with alkali metal or by doping.^54–58^ The phenomenon was explained by the transfer of electron from the s orbital of the alkali metal to the d orbital of the transition metal, which attributes to the destabilization of the 1T phase. In other cases, a different evolution from 2H-TMD to a surface alloy monolayer, with a clear boundary between the two phases due to the partial phase transition, has been observed.^42,59^ The transitions of 1T or the 2H structure of the PtSe_2_ have been reported using different synthesis methods,^21,26,30^ but to our knowledge, no phase transition to a surface alloy monolayer has been reported. In our present study, we highlighted through systematic analysis a transition from 2H-PtSe_2_ phase to a Pt_2_Se alloy monolayer. The PtSe_2_ firstly displayed a 2H trigonal prismatic phase, in which the Pt atom was prismatically bonded to six Se atoms after annealing at 400–500 °C. Interestingly, the (4 × 4) structure is nicely observed by the HR-STM, where a moiré pattern with honeycomb arrangement is seen, in perfect agreement with the LEED investigations. The similarity of the Se3d core level in the 300–500 °C range indicated the same architectural properties in the PtSe_2_ films. Further annealing, inducing desorption of Se atoms, leads to formation of a Pt_2_Se alloy monolayer characterized by its (√3 × √3)R30° superstructure. This alloy phase is known to develop commonly in a large number of systems, like Cu_2_Te/Cu(111),^9^ Ag_2_Bi/Ag(111),^60,61^ Cu_2_Bi/Cu(111),^62^ Ag_2_Sb/Ag(111),^63^ and others. Such a phase transition induced the significant altering of the chemical bonding state of the Pt and Se. This is reflected in the Pt4f and Se3d core level spectra, in both of which the previously multi-components spectrum turned into a single dominant component spectrum, leaving just mainly the bulk Pt and Se^2+^ features, respectively. At higher temperature, another surface structure occurs and exhibits a (2 × 2) superstructure observed by LEED and confirmed by LT-STM. This latter is characterized by a relatively high density of defects.

## V. Conclusion

In summary, we have highlighted a new approach for the preparation of two-dimensional TMD monolayer, in particular for the PtSe_2_ system. It consists in chemical deposition upon immersion of Pt(111) surface into the Na_2_Se aqueous solution. Although this method is rather simple, it provides high quality and well-ordered films on a large scale. A carbon- and oxygen-free surface is obtained after a moderate annealing procedure. Depending on the annealing temperature of the sample, the surface undergoes different phase transitions. LEED investigations revealed a transition from (4 × 4) to (√3 × √3)R30° and then to a (2 × 2) superstructures, which is also confirmed by STM showing structural transformation from 2H-PtSe_2_ to Pt_2_Se alloy monolayer. Analysis of the XPS data proves the presence of different chemical environments and the structural transitions influence directly the electronic structure. This work provides an alternative method for the fabrication of large-scale 2D transition metal dichalchogenide films, as well as single alloy layer.

## Conflicts of interest

The authors declare no conflicts of interest.

## Supplementary Material
